# Neural substrates underlying fear-evoked freezing: the periaqueductal grey–cerebellar link

**DOI:** 10.1113/jphysiol.2013.268714

**Published:** 2014-04-22

**Authors:** Stella Koutsikou, Jonathan J Crook, Emma V Earl, J Lianne Leith, Thomas C Watson, Bridget M Lumb, Richard Apps

**Affiliations:** 1 School of Physiology and Pharmacology, Medical Sciences Building, University of BristolBristol, BS8 1TD, UK; 2 School of Biological Sciences, University of BristolBristol, BS8 1UG, UK

## Abstract

The central neural pathways involved in fear-evoked behaviour are highly conserved across mammalian species, and there is a consensus that understanding them is a fundamental step towards developing effective treatments for emotional disorders in man. The ventrolateral periaqueductal grey (vlPAG) has a well-established role in fear-evoked freezing behaviour. The neural pathways underlying autonomic and sensory consequences of vlPAG activation in fearful situations are well understood, but much less is known about the pathways that link vlPAG activity to distinct fear-evoked motor patterns essential for survival. In adult rats, we have identified a pathway linking the vlPAG to cerebellar cortex, which terminates as climbing fibres in lateral vermal lobule VIII (pyramis). Lesion of pyramis input–output pathways disrupted innate and fear-conditioned freezing behaviour. The disruption in freezing behaviour was strongly correlated to the reduction in the vlPAG-induced facilitation of α-motoneurone excitability observed after lesions of the pyramis. The increased excitability of α-motoneurones during vlPAG activation may therefore drive the increase in muscle tone that underlies expression of freezing behaviour. By identifying the cerebellar pyramis as a critical component of the neural network subserving emotionally related freezing behaviour, the present study identifies novel neural pathways that link the PAG to fear-evoked motor responses.

## Introduction

Responding adequately to events that threaten an animal's survival requires close interaction between sensory, autonomic and somatic–motor systems in order to generate the appropriate, integrated behavioural response. Fear is a vital response to danger and elicits characteristic patterns of defensive behaviour, including freezing, which are conserved across all mammalian species. There is also a growing consensus that understanding the neural circuits that support the behavioural and physiological responses to fear in animals is a fundamental step towards understanding emotional disorders in humans (Johansen *et al*. 2011; Parsons & Ressler, 2013). The periaqueductal grey (PAG) lies at the heart of the defence–arousal system and is critically involved in survival behaviours (LeDoux, 2012). Lesions of its ventrolateral sector (vlPAG) reduce the freezing component of a Pavlovian conditioned aversive response (LeDoux *et al*. 1988; Vianna *et al*. 2001b), while expression of conditioned freezing is associated with increased vlPAG neuronal activation (Carrive *et al*. 1997).

Attention to date has focused on neural pathways underlying autonomic and sensory consequences of vlPAG activation, and polysynaptic descending paths that modulate autonomic outflow and sensory processing at the level of the spinal cord are well documented (Lovick & Bandler, 2005). By contrast, much less is known about the neural pathways that link vlPAG activity to distinct patterns of motor response associated with survival behaviours such as freezing. This is a significant gap in our knowledge given the survival value of initiating, adapting or maintaining co-ordinated motor responses in aversive or threatening situations.

In order to elicit freezing behaviour the vlPAG must engage spinal motor circuits. Since freezing behaviour can be elicited from the vlPAG in decerebrate animals, descending projections are necessary and sufficient to elicit such responses (Keay & Bandler, 2001). While both direct (Mouton & Holstege, 1994) and indirect routes from PAG, via the brainstem, to the spinal cord have been described (Mantyh, 1983), some anatomical evidence for projections from the PAG to the cerebellum has also been noted in cat (Dietrichs, 1983) and human (Sillery *et al*. 2005). The PAG also has direct anatomical projections to the lateral reticular nucleus, a major pre-cerebellar nucleus (Roste *et al*. 1985) as well as projections to the inferior olive (Swenson & Castro, 1983a, *b*1983b; Rutherford *et al*. 1984; Watson *et al*. 2013). The latter is the sole source of climbing fibre afferents to the cerebellum. In addition, activation of vlPAG can have a profound influence on transmission in ascending sensorimotor pathways that terminate as climbing fibres in the cerebellar cortex (Cerminara *et al*. 2009). Thus, multiple routes exist by which the PAG could engage with cerebellar circuits to control the motor aspects of defence behaviours.

There is a growing body of evidence to support the role of the cerebellum, and in particular its vermal compartment, in emotionally related defence behaviours. This has stemmed in part from the observation of a correlation between cerebellar pathology and abnormal emotionally related behaviour both in humans and animals (Berntson *et al*. 1973; Reis *et al*. 1973; Berntson & Schumacher, 1980; Turner *et al*. 2007; Hopyan *et al*. 2010). However, the neural pathways that link the cerebellum with the defence–arousal system are poorly understood.

In the present study we have tested the hypothesis that fear-evoked freezing associated with vlPAG activation is cerebellar dependent. Using *in vivo* electrophysiological field potential mapping techniques we have identified a potent physiological connection between vlPAG and the posterior lobe of the cerebellar cortex, localised to cerebellar vermal lobule VIII (pyramis). A combination of behavioural and lesioning techniques identify this novel pathway as part of a neural network involved in the expression of fear-evoked freezing behaviour. In addition, we also address an important conflict in the literature regarding whether or not vlPAG activation causes an increase in muscle tone during fear-evoked freezing (Widdowson *et al*. 1986; Vianna *et al*. 2001a; Walker & Carrive, 2003). We provide the first direct evidence that vlPAG activation can increase muscle tone. Lesions of input–output pathways of the pyramis disrupt vlPAG-induced increases in muscle tone and disrupt conditioned (learned) and innate fear-evoked freezing behaviour, but have no detectable effect on general motor performance, nor on the affective state of the animal. Taken altogether this indicates that a highly specific, basic survival behaviour known to be controlled by the PAG is dependent on the cerebellar pyramis.

## Methods

### Ethical approval

All animal procedures were performed in accordance with the UK Animals (Scientific Procedures) Act 1986 and associated guidelines. Male adult Wistar rats (250–300 g; Charles River, Margate, Kent UK) were used throughout this study. They were housed under normal environmental conditions (∼20°C and 45–65% humidity), on a 12 h dark–light cycle and provided with food and water *ad libitum*.

### General surgical procedures

For mapping evoked cerebellar responses, animals were anaesthetised with sodium pentobarbitone (Sigma, Gillingham, Dorset UK; 60 mg kg^−1^). For all H-reflex experiments, animals were anaesthetised with Alfaxan (alphaxalone; 25 mg kg^−1^ h^−1^; i.v.; Jurox, Malvern Link, Worcestershire UK), which has been extensively used in our previous studies into vlPAG descending control on spinal reflexes because of the stable plane of general anaesthesia it can produce (McMullan & Lumb, 2006; Leith *et al*. 2010). In every case, body temperature was maintained and regulated within physiological limits (∼37°C). Animals were placed in a stereotaxic frame and where appropriate the posterior cerebellum was exposed to allow access to the pyramis; a small-bore hole was made for a dorsal approach to PAG (for further details see section below). In recovery experiments, intraperitoneal anaesthesia 1.8 ml kg^−1^ of a mixture of ketamine (Vetalar, Boehringer Ingelheim Vetmedica Inc., Missouri USA: 0.6 ml) and medetomidine (Domitor, Pfizer, Surrey UK: 0.25 ml) made up in 1 ml of saline (0.9%) was used. The overlaying layers of muscle and skin were closed in layers using a suture (Vicryl 4–0, Ethicon, Wokingham, UK) and the animals were allowed to recover for 4 days post-operatively prior to any other experimental testing.

### Recording of cerebellar cortical-evoked field potentials

In seven animals extracellular field potentials were recorded from the cerebellar cortical surface using a low impedance silver ball electrode. Responses were evoked using a bipolar stimulating electrode (SNE-100, Harvard Apparatus, Kent, UK) implanted stereotaxically into vlPAG (single or dual (1 kHz) square wave electrical pulse 0.2 ms, intensity range 120–400 μA). Evoked responses were recorded differentially between the cerebellar cortical electrode and an indifferent (Ag–AgCl disc) electrode placed in the bone margin lateral to the cerebellar exposure. Responses were amplified (× 2k) and filtered (30 Hz to 2.5 kHz, Neurolog system, Digitimer Ltd, Welwyn Garden City, UK), with any 50 Hz electrical interference removed by a Humbug device (QuestScientific, distributed by Digitimer Ltd). The signal was sampled at 20 kHz using a CED 1401*plus* A/D converter (Cambridge Electronic Design (CED), Cambridge, UK) and analysed off-line (peak-to-peak amplitude and latency to onset of the initial rising phase of individual fields) using Spike2 software (CED). Recording positions were visually plotted onto a standard anatomical map of the cerebellar cortex (Larsell, 1952). At the end of each experiment an electrolytic lesion (20 mA, negative square wave pulse, 10–20 s) was produced by the stimulating electrode in order to aid post-mortem histological identification of stimulus location in PAG.

### Single unit recording of complex spike activity

A glass-coated tungsten microelectrode (Merrill & Ainsworth, 1972) was inserted orthogonal to the surface of cerebellar lobule VIII (pyramis) and single unit Purkinje cell activity recorded immediately subjacent to the site where the largest field potentials were found on the cerebellar cortical surface. Purkinje cells were identified by the presence of complex spike activity. Single-unit neuronal activity was amplified (×10k) and filtered (500 Hz to 10 kHz; Neurolog) before being sampled at 10 kHz via a 1401*plus* (CED) and analysed off-line using Spike2 software (CED e.g. construction of peri-event time histograms).

### Behavioural studies

Animals (total *n *= 22) were acclimatised for 1–3 days to all behavioural contexts, apart from the open field test and the elevated plus maze. Fear conditioning (*n *= 22) and testing for freezing (see below) took place in two different contexts (A and B, respectively). The Skinner box (Med Associates Inc., St Albans, VT, USA) and its floor were cleaned thoroughly with 70% ethanol after every session. On days 1–3, animals were acclimatised for 5 min each day to context A. On day 4, in context A, rats were exposed to an auditory cue (conditioned stimulus, CS)–footshock (unconditioned stimulus, US) fear-conditioned protocol. This involved seven trials (30 s inter-trial interval) of paired CS (1 kHz auditory tone, 75 dB, 10 s duration) and US presentations (Sacchetti *et al*. 2004).

### Microinjections of cholera toxin subunit b–saporin

On day 5, animals were anaesthetised (see above) and microinjections (60 nl each) of the lesioning tracer, cholera toxin subunit b (CTb)–saporin (Advanced Targeting Systems, San Diego, CA, USA; *n *= 12), CTb-only, or saporin-only (Sham experiments; *n* = 10) were made into the superficial cortex of the pyramis (perpendicular to the surface at a depth of approximately 250–300 μm) with a glass micropipette (tip diameter 8–12 μm) attached to a custom-made pressure device. In initial experiments we made a series of four equally spaced injections across the width of the pyramis (*n* = 6). In all subsequent experiments, two injections were made, each one just medial to the paravermal vein on either side of the midline (*n* = 16), where electrophysiological experiments identified the largest field potentials that were evoked by vlPAG stimulation (see Fig.1*A*). For subsequent analysis of fear-evoked freezing behaviour these two groups of animals (four *versus* two microinjections) were pooled as no significant difference was observed in freezing behaviour (Kruskal–Wallis; *P* > 0.05).

**Figure 1:**
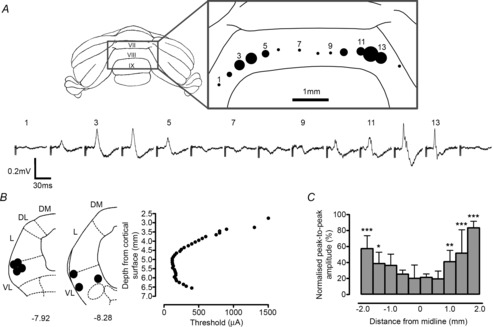
Cerebellar cortical fields evoked by vlPAG stimulation *A*, schematic diagram of vermal lobule VIII (pyramis) displaying the distribution and amplitude of field potentials evoked by electrical stimulation of the vlPAG in a typical experiment. Expanded region of the cerebellar vermis indicates recording site loci and each filled circle displays the relative size of the peak-to-peak amplitude of the evoked field potential for the corresponding numbered sequence of waveform averages displayed below (each trace average of 3 trials). *B*, left-hand panel shows on a standard map of the left PAG sites of electrical stimulation where cerebellar cortical field potentials were evoked in all available cases (*n* = 7). Coordinates are relative to bregma (DM, dorsomedial; DL, dorsolateral; L, lateral; VL, ventrolateral). The right-hand panel plot is an example case of thresholds for evoking a cerebellar cortical field at the optimum site of recording, as a function of dorsoventral depth. Measurements were made relative to the surface of the brain. Lowest threshold values correspond to vlPAG. *C*, pooled data showing average peak-to-peak amplitude of evoked field potentials as a function of mediolateral recording position on the surface of the pyramis. Data are normalised to the largest value recorded in each case (100%) and are presented as median and interquartile range (*n* = 7, *P < 0.05, **P < 0.01, ***P < 0.001, Kruskal–Wallis test with Dunn's *post hoc* tests compared to values obtained at recording sites closest to the midline).

### Fear-conditioned testing

After days 9–11 (4–6 days post-surgery), to allow neuronal degeneration in cerebellar cortex and precerebellar nuclei, including the inferior olive (Pijpers *et al*. 2008), each animal (*n *= 22) was placed in the Skinner box, with context B (different from Context A), and after 3 min to acclimatise they were presented with 7 × CS. The freezing response was measured as a percentage of time immobile during the 7 × 10 s duration of the CS and the 7 × 30 s duration of the inter-trial interval. Freezing was defined as the cessation of all movements except those associated with respiration and eye movements and was typically characterised by crouching postures (Blanchard & Blanchard, 1969). Animals included in this set of experiments showed baseline exploratory behaviour indistinguishable from that of normal animals.

### Open field test

This is a test widely used in rodent behavioural studies to evaluate general motor activity and levels of anxiety evoked by a novel environment (Cryan & Sweeney, 2011). Each animal (CTb–saporin, *n* = 10; Sham, *n* = 8) was placed at the perimeter of the open field (circular arena, diameter 75 cm, wall height 85 cm) and allowed to explore freely for 10 min. Behaviour was recorded by a video camera and blinded analysis was performed offline. Exploratory behaviour (motility score) was assessed by calculating the total number of grid squares crossed in the 10 min time period. Measuring the latency to enter the centre of the arena assessed anxiety.

### Elevated plus maze

This is a test widely used to monitor levels of anxiety in rodents (Pellow *et al*. 1985). The maze consisted of two open arms, 10 cm wide and 50 cm in length, connected perpendicular to two enclosed arms of equal dimensions with a square centre region. The enclosed arms had opaque walls 30 cm in height and the maze was elevated 1 m above the floor. The open arms had 1 cm curbs along the edges to enable better grip when the animals head-dipped over the edges. Each animal (CTb–saporin, *n* = 10; Sham, *n* = 8) was placed in the centre region facing a closed arm and allowed to explore for 5 min. An overhead camera attached to a PC recorded the movements of the rats and blinded scoring was completed offline. Anxiety was assessed by calculating the percentage time spent in the open arms, percentage entries to the open arms, and number of entries to the enclosed arms (Pellow *et al*. 1985).

### Vertical grid test

This behavioural test assesses general co-ordination, orientation and also muscle tone in terms of grip strength (Joyal *et al*. 2001). On the day of the test each animal (CTb–saporin, *n* = 12; Sham, *n* = 10) was positioned on the vertical grid (wire net 1 square cm^−1^) 1 m above the floor, facing downward. Latency to turn and face upward was measured during two trials on a single day of testing. The inter-trial interval was 10 min and the cut-off time 120 s per trial.

### Foot gait analysis

This test evaluates limb movements in order to assess changes in gait during locomotion (Seoane *et al*. 2005). Hind- and forepaws were marked with non-toxic ink and the rats (CTb–saporin, *n* = 11; Sham, *n* = 8) then walked across chart paper within a narrow box (10 cm width × 1 m length), leaving a permanent record of their footprints. Step width was measured to detect any signs of ataxia.

### Cat odour test

This is a well-established method to evoke unconditioned fear behaviour in rats (Dielenberg & McGregor, 2001). Animals (total *n* = 12, previously used in the fear conditioning protocol) were acclimatised for 5 min to a recording cage dissimilar to the home cage before introducing two clean filter papers at the top corner of the cage for an additional 10 min. At least 24 h after acclimatisation, and following CTb–saporin lesions (*n* = 6) or Sham treatment (*n* = 6) as described above, each rat was returned to the recording cage and after 5 min two filter papers impregnated with cat odour were placed in the top corner of the cage. Behavioural recordings were then made for a further 10 min. Video recordings were made using a PC camera and offline analysis of freezing and risk assessment behaviours (Blanchard *et al*. 1990; Misslin, 2003) was conducted in a blinded manner.

### H-reflex recordings

In anaesthetised animals (see above) a pair of stimulating electrodes (25G) was inserted subcutaneously between the Achilles’ tendon and the distal tibial nerve of the left hindlimb. Constant current 50 μs square wave pulses were delivered at 3 s intervals. A pair of intramuscular stainless steel recording electrodes (0.075 mm in diameter Teflon-coated; Advent Research Materials, UK), were inserted into the ipsilateral plantaris muscle to record evoked EMG activity (M-wave and H-reflex) in response to low intensity electrical stimulation of the nerve (Mattsson *et al*. 1984; Gozariu *et al*. 1998). In all cases the stimulus intensity was adjusted so that it was submaximal and the H-reflex amplitude was larger than the M-wave. The responses were amplified (× 2k) and filtered (50 Hz to 5 kHz; Neurolog system) before being captured via a 1401*plus* A/D device (CED). The individual H-reflex and M-wave peak-to-peak amplitudes evoked by each stimulus were measured using Spike2 software (CED). M-wave and H-reflex responses were recorded before and after chemical stimulation of vlPAG (see below). The mean of five responses in each period: (i) prior, (ii) immediately after, and (iii) 10 min after microinjections of dl-homocysteic acid (DLH; an excitatory amino acid, see also below) were averaged and statistically compared to determine any influence of the vlPAG (*n* = 16) on H-reflex amplitude. In all cases the H-reflex data were normalised with respect to the M-wave (H:M ratio).

### Neuronal activation of the periaqueductal grey

Glass micropipettes were positioned in caudal vlPAG under stereotaxic guidance (Paxinos & Watson, 2005) at approximately 7.6–8.3 mm caudal to bregma, 0.8–0.9 mm lateral to midline and 5.25–5.3 mm deep to the cerebral cortical surface. Micropipettes were filled with DLH (50 mm; Sigma) in a solution containing pontamine sky blue dye (the latter to recover the location of injection sites post-mortem). Chemical stimulation using DLH excites neuronal somata and dendrites but not axonal fibres of passage (Goodchild *et al*. 1982; Lipski *et al*. 1988). This method of stimulation has been used extensively in acute (Koutsikou *et al*. 2007; Waters & Lumb, 2008; Cerminara *et al*. 2009) and awake behaving rats (Morgan & Carrive, 2001) to study descending control by the PAG. Consistent with targeting vlPAG in the anaesthetised animal, DLH injections (60–80 nl) typically evoked decreases in mean arterial blood pressure, which enabled us to confirm physiologically the neuronal activation of vlPAG at the time of the experiment. This was verified post-mortem by histological recovery of injection sites (see below).

### Subtotal lesions of the inferior olive

In some of the H-reflex experiments above, following testing of the effects of vlPAG activation on H-reflex amplitude, we either (i) intraperitoneally administered the neurotoxin *trans*-crotononitrile (TCN; 750 mg kg^−1^ in corn oil; *n* = 4) or (ii) intraperitoneally administered vehicle (Sham; ∼0.4 ml corn oil; *n* = 5). Three hours after each intervention, neuronal activation of vlPAG on H-reflex amplitude was tested as described above.

### Histological preparation

At the end of every experiment, animals were perfusion fixed transcardially, under deep anaesthesia (Euthatal, 200 mg ml^−1^, Merial Animal Health Ltd, Harlow, UK), with 300 ml of saline followed by 400 ml paraformaldehyde (4% in 0.1 m phosphate buffer). Brains were removed and post-fixed overnight. The tissue was then transferred to 30% sucrose for at least 24 h. The cerebella and brainstems were then removed and embedded in blocks of gelatin (type A, 300 bloom, Sigma).

### Periaqueductal grey injection sites

Coronal sections (60 μm) of the midbrain were cut on a Peltier freezing microtome and collected in 0.01 m phosphate buffer (PB). Prior to sectioning, all tissue was marked to aid orientation. Sections were mounted onto microscope slides and viewed with a Zeiss Axioskop 2+ microscope. Injection sites were identified by the location of the pontamine sky blue dye mark with reference to a stereotaxic atlas (Paxinos & Watson, 2005).

### Cholera toxin subunit b immunostaining

Coronal sections (50 μm) of gelatin-embedded cerebella and brainstems were cut on a Peltier freezing microtome and collected in 0.01 m PB. CTb immunostaining was carried out using a standard immunostaining protocol. Free-floating sections were processed for anti-CTb immunostaining using a primary polyclonal goat anti-CTb antibody (List Biological Laboratories, Campbell, CA, USA; 1:15,000) and a secondary biotinylated donkey anti-goat antibody IgG (Sigma; 1:500). The sections were subsequently incubated in extravidin peroxidase (Sigma; 1:1000) in order to allow visualisation using the nickel-intensified 3,3-diaminobenzidine (DAB) reaction staining method. Two series were mounted onto microscope slides. One series was used for analysis, the second used as a spare. Sections were viewed with a Zeiss Axioskop 2+ microscope and the distribution of retrograde labelling in the inferior olive (cell bodies and lysed cells) was mapped onto standard horizontal maps of the inferior olive (Pardoe & Apps, 2002).

### Data analysis and statistics

The data are expressed as mean ± SEM and *n* denotes the number of rats used. Statistical analysis was performed using GraphPad Prism 4.0. Behavioural comparisons between groups were made using the Mann–Whitney test. Electrophysiological data were analysed with Student's *t* test, Kruskal–Wallis test, or one-way ANOVA test, as appropriate. In all tests *P* < 0.05 was taken to represent a statistically significant difference.

## Results

### Physiological evidence for a PAG–cerebellar link

As a first step in identifying a possible neural substrate responsible for mediating the vlPAG influence on spinal motor output that underlies fear-evoked freezing behaviour, we used *in vivo* electrophysiological mapping techniques to chart the pattern of neural connectivity that links vlPAG with the cerebellum. Figure1*A* shows representative data from a single experiment in a barbiturate-anaesthetised rat in which electrical microstimulation of vlPAG on one side of the brain (Fig.1*B***)** evoked field potentials on the cerebellar cortical surface. Consistent with the experiments as a whole, the evoked responses were largest in the vermis of lobule VIII (pyramis; *n *= 7; *P *< 0.001). The largest fields (maximum size of peak-to-peak amplitude ranged in individual experiments from 0.28 to 0.72 mV) were present bilaterally, predominantly localised to lateral aspects of the pyramis, just medial to the paravermal veins, with a mean onset latency of 15.1 ± 0.8 ms (Fig.1*A* and *C***)**. Whilst a complete survey of the cerebellar surface was not possible because of blood sinuses restricting access *in vivo*, in the same animals responses evoked by electrical microstimulation of vlPAG in other areas of the posterior cerebellar cortex, including other vermal lobules (data not shown) and regions of the paravermis (some data shown in Fig.1*A*), when present were invariably smaller in size than those evoked in the pyramis.

The evoked responses in the pyramis displayed features typical of climbing fibre field potentials, including their waveform (Figs1*A* and 2*D*), their stimulus–response characteristics (Fig.2*A*), and their pattern of response to a paired pulse test (Fig.2*B*). Although we cannot exclude the possibility that part of the responses presented in Figs. 1 and 2 are mossy fibre related, it is likely that they were due mainly to transmission in the olivocerebellar climbing fibre system. This is because barbiturate anaesthesia is known to severely depress transmission in cerebellar cortical parallel fibres, and as a consequence Purkinje cells respond only weakly to mossy fibre inputs (Gordon *et al*. 1973). This is supported by results from single unit studies in which complex spike activity of individual Purkinje cells was recorded at sites in lateral pyramis immediately subjacent to where the largest field potentials were evoked on the cerebellar cortical surface. When tested, electrical microstimulation of vlPAG elicited complex spikes at a latency similar to the evoked fields (Fig.2*C* and *D*).

**Figure 2:**
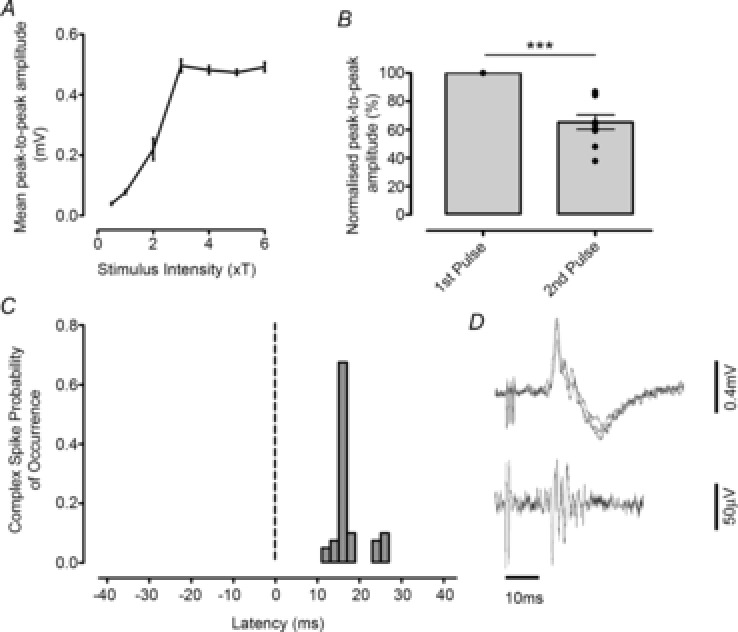
Ventrolateral PAG–olivocerebellar link *A*, example case showing peak-to-peak amplitude of cerebellar cortical field potentials evoked by ventrolateral (vl) PAG electrical stimulation as a function of stimulus intensity, relative to threshold (*T*) for evoking a just detectable cerebellar response. Data are presented as means of 6 trials ± SEM. *B*, data from a paired pulse test carried out during a single experiment, displaying average peak-to-peak amplitude of cerebellar cortical field potentials evoked by a second stimulus delivered 30 ms after an initial stimulus. Data are normalised to the amplitude of the first field potential and both stimulus pulses are delivered to vlPAG at supramaximal intensity. Data are presented as mean (±SEM) of 10 individual trials (***P < 0.001, one sample *t* test). *C*, an example peristimulus time histogram of complex spike occurrence following vlPAG electrical stimulation (time zero, vertical dashed line) for an individual case. *D*, example traces of cerebellar cortical surface field potential (upper trace, overlay of 3 consecutive trials) and complex spike activity (lower trace, single sweep) in a Purkinje cell located in the Purkinje cell layer immediately subjacent to the surface recording. Both responses evoked by electrical stimulation at the same vlPAG site and traces aligned to first stimulus artefact.

The field potential mapping experiments therefore demonstrate that a strong physiological link exists between vlPAG and the cerebellar pyramis and that, under the present experimental conditions, the pathway identified is likely to be relayed mainly, if not exclusively, via the olivocerebellar system. However, it should be emphasised that this does not exclude the possibility that other (mossy fibre) pathways may also exist.

### Role of cerebellar pyramis in fear-evoked freezing behaviour

If the cerebellar pyramis is a key supraspinal structure within a chain of connections that link the vlPAG to the expression of the motor aspects of fear-conditioned freezing behaviour, then it follows that targeted lesion of this cerebellar region should disrupt such behaviour. This hypothesis was tested directly in animals (*n *= 12) trained in an auditory cue-conditioned fear protocol (conditioned (CS)–unconditioned (US) association) followed by microinjections into the pyramis of the neurotoxin-labelled tracer CTb–saporin (Llewellyn-Smith *et al*. 2000). An additional 10 animals were also trained in the fear CS–US protocol but subsequently received either CTb-only (*n* = 7) or saporin-only (*n* = 3) microinjections into the pyramis (Sham animals). The data for both groups of Sham animals are pooled because they displayed no significant differences in fear-evoked freezing behaviour (*P* = 0.55, Mann–Whitney test).

When tested for retrieval of the conditioned freezing response, CTb–saporin animals, in contrast to Sham animals, showed a statistically significant reduction in duration of freezing behaviour to the CS tone only (by 31.3 ± 2.9%; *P* < 0.001; Fig.3*A* and Video S1 in the online Supporting information). Both CTb–saporin and Sham animals showed normal acoustic startle responses (hand clapping, data not shown) and were also tested in a battery of general motor and anxiety-generating behavioural tasks (elevated plus maze, open field, vertical grid test, foot gait analysis). The performance of both CTb–saporin and Sham animals in all these tasks was indistinguishable from one another (*P* > 0.05, Fig.4*A–H*).

**Figure 3:**
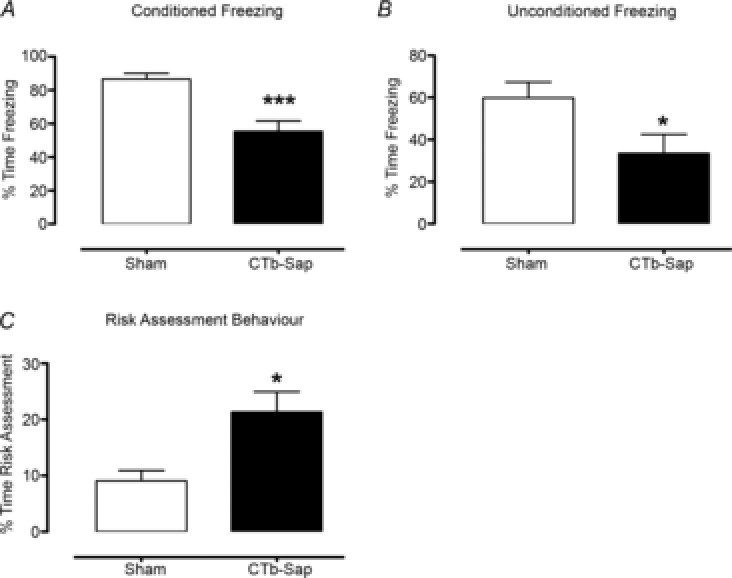
CTb–saporin lesions of the cerebellar pyramis reduce fear-induced freezing behaviour *A*, CTb–saporin-treated rats (CTb-Sap; *n* = 12) displayed a significant reduction in duration of freezing response (expressed as a % of total time) in comparison to Sham-treated rats (*n* = 10), during exposure to a conditioned auditory tone previously associated with an aversive footshock (***P < 0.001, Mann–Whitney test; see also Video S1 in online Supporting information). *B*, some of the CTb–saporin-treated rats (CTb-Sap; *n* = 6) were also exposed to an unconditioned cat-odour stimulus. By comparison to Sham-treated rats (*n* = 6) they displayed a significant reduction in duration of freezing response (*P < 0.05, Mann–Whitney test). *C*, in the same rats shown in *B* there was a significant increase in risk assessment behaviour of CTb-Sap-treated rats (*n* = 6) in comparison to Sham-treated rats (*n* = 6), during exposure to the cat-odour stimulus (*P < 0.05, Mann–Whitney test; see also Video S2 in online Supporting information).

**Figure 4:**
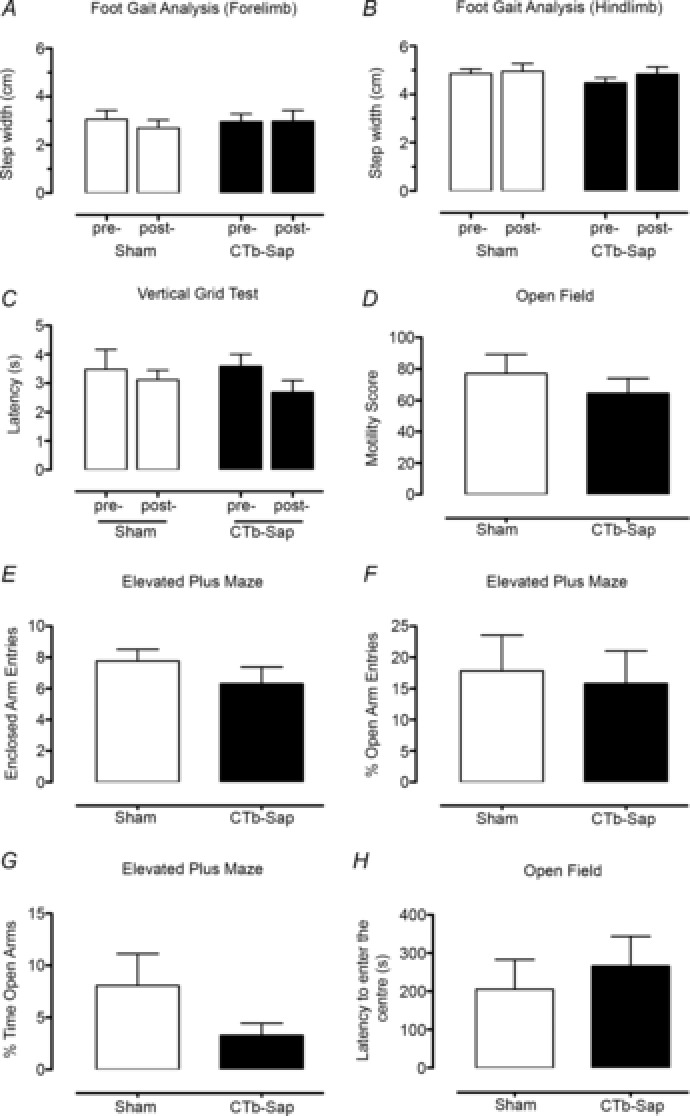
CTb–saporin (CTb-Sap) lesions of the cerebellar pyramis have no detectable effect on general motor- and anxiety-related performance *A* and *B*, foot gait analysis revealed no statistically significant changes in fore- and hindlimb step width of CTb-Sap (filled bars, *n* = 11) compared to Sham-treated rats (open bars, *n* = 8; *P* > 0.05, Mann–Whitney test), pre- and post-treatment. *C*, vertical grid test performance revealed no statistically significant change in muscle tone, balance and general co-ordination in CTb-Sap (filled bars, *n* = 12) compared to Sham-treated rats (open bars, *n* = 10, *P* > 0.05, Mann–Whitney test), pre- and post-treatment. *D* and *E*, locomotor activity was evaluated in both the open field and the elevated plus maze. No statistically significant differences were found between CTb-Sap (filled bars, *n* = 10) and Sham-treated rats (open bars, *n* = 8) in both tests (*P* > 0.05, Mann–Whitney test). *F–H*, anxiety-related behaviour was evaluated in both the open field and the elevated plus maze. No statistically significant differences were found between CTb-Sap (filled bars, *n* = 10) and Sham-treated rats (open bars, *n* = 8) in both tests (*P* > 0.05, Mann–Whitney test).

The significant decrease in fear-conditioned freezing behaviour in CTb–saporin animals may be due to an inability to retrieve the associative memory. We tested this possibility by exposing some of the same animals to an unconditioned (innate) fear protocol (exposure to cat odour, see Methods). The amount of innate freezing was significantly reduced in CTb–saporin (*n *= 6) rats compared to Sham animals (*n *= 6; *P *< 0.05; Fig.3*B* and Video S2 in the online Supporting information). Typically, freezing was replaced by risk assessment behaviour (Sham *vs*. CTb–saporin, *P *< 0.05, Fig.3*C* and Video S2), which included stretched attend movements oriented towards the threat stimulus, together with directed sniffing (Blanchard *et al*. 1990; Misslin, 2003). The directed sniffing suggests that the lesioned rats were still able to detect the cat odour.

### Cerebellar pyramis links ventrolateral PAG with spinal motor circuits

In order to monitor the effects of vlPAG activation on spinal motor activity in the anaesthetised preparation we recorded changes in the peak-to-peak amplitude of the H-reflex, which is an indirect but reliable measure of α–motoneurone excitability and can therefore provide a ‘readout’ of muscle tone (Lance, 1980; Schieppati, 1987). Figure5*A* illustrates a typical example in which neuronal excitation in vlPAG with DLH in an alphaxalone-anaesthetised rat increased the amplitude of the H-reflex response relative to baseline (Fig.5*A*, pre-PAG *vs*. PAG**)**. On average, neuronal activation of vlPAG significantly increased the peak-to-peak amplitude of the H-reflex by 45.2 ± 11% (*n *= 16; *P *< 0.001; Fig.5*B*). The H-reflex magnitude returned to baseline levels within a 10 min period following vlPAG activation (Fig.5*B*, post-PAG). The M-wave remained similar in amplitude throughout the experiment (*P *> 0.5; Fig.5*A* and *B*), indicating that the effects on the H–reflex occurred centrally rather than being due to any peripheral changes in stimulus efficacy (Boorman *et al*. 1996). Post-mortem histological reconstruction confirmed that the microinjections of DLH were located within vlPAG (filled circles, *n* = 13; Fig.5*C*). Overall, these data therefore provide evidence that spinal α-motoneurone excitability, and by inference muscle tone, can be strongly facilitated by the vlPAG.

**Figure 5:**
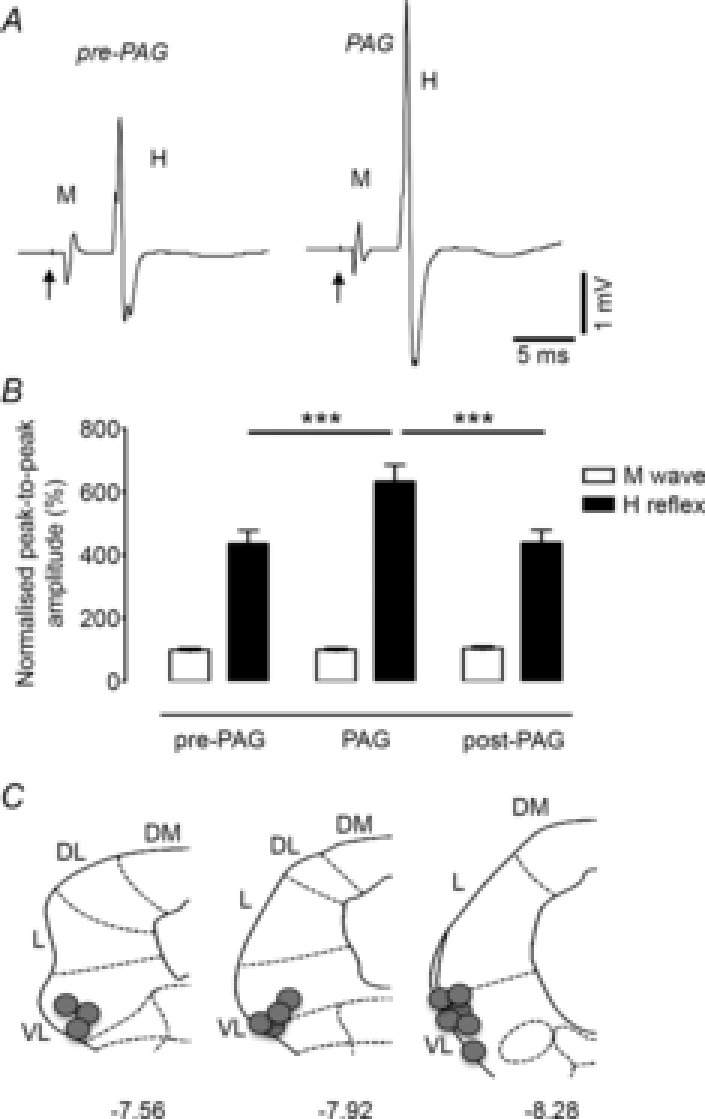
Ventrolateral PAG activation facilitates the peak-to-peak amplitude of the H-reflex *A*, typical examples of averaged M-wave (M) and H-reflex (H) evoked by electrical stimulation of the ipsilateral tibial nerve. Each example consists of five consecutive responses averaged before (pre-PAG) and during (PAG) vlPAG chemical excitation with dl-homocysteic acid (DLH). Arrows indicate onset of electrical stimulus. *B*, peak-to-peak amplitude of M-wave and H-reflex before (pre-PAG), during (PAG) and after (post-PAG) vlPAG neuronal activation by DLH. Data are normalised to the M-wave average (*n* = 16, ***P < 0.001, one-way ANOVA with Bonferroni's *post hoc* test). *C*, standard transverse maps of the left PAG to show injection sites of DLH in the vlPAG (*n *= 13; filled circles), recovered from 13 of the above animals, from which the effects of vlPAG activation on peak-to-peak amplitude of M-wave and H-reflex were tested. The coordinates are relative to bregma (DM, dorsomedial; DL, dorsolateral; L, lateral; VL, ventrolateral).

To determine whether the link between vlPAG and pyramis plays an important role in PAG-mediated effects on spinal α-motoneurone excitability, some of the animals (*n* = 6) used in the behavioural studies described above were also used in a terminal experiment (under alphaxalone anaesthesia, in keeping with our initial H-reflex experiments) in order to examine vlPAG-induced changes in H-reflex amplitude. A week after CTb–saporin was microinjected into the pyramis, group analysis showed that DLH activation of vlPAG failed to exert a significant facilitatory effect on H-reflex amplitude (*P* > 0.05, Fig.6*A*). For individual animals, changes in freezing behaviour were plotted as a function of vlPAG-induced changes in H-reflex amplitude (Fig.6*B*). Consistent with H-reflex excitability being a reliable monitor of muscle tone underlying freezing behaviour, a strong positive correlation was found between the two parameters (*n* = 6; Pearson correlation coefficient; *r*^2^ = 0.75, *P* < 0.05).

**Figure 6:**
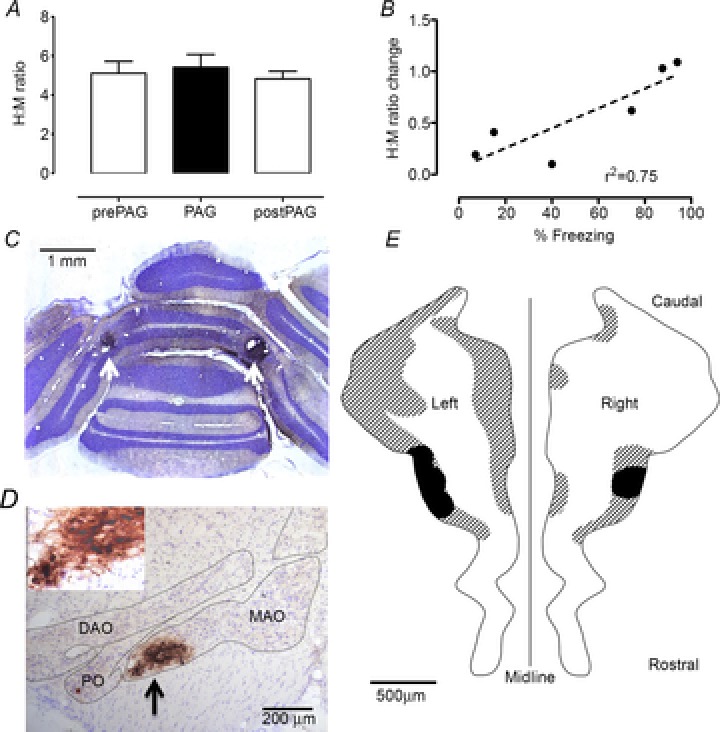
Neurotoxin tracer lesion of connections of the pyramis abolishes the facilitatory influence of vlPAG activation on H-reflex amplitude *A*, group data showing that microinjections of CTb–saporin into superficial cortical layers of cerebellar vermal lobule VIII (pyramis) prevent the facilitatory influence of vlPAG activation on mean H-reflex amplitude (*n* = 6; *P* > 0.05). *B*, fear-evoked freezing behaviour is positively correlated (*r*^2^ = 0.75; *P* < 0.05; *n* = 6) to changes in H-reflex amplitude (H:M ratio change) evoked by chemical excitation of the ventrolateral PAG. *C*, example case showing location of injection sites (arrows) within the pyramis. Cerebellar section viewed in the transverse plane. *D*, example case (different from *C*) showing location of cells retrogradely labelled with CTb in the medial accessory olive (MAO). Brainstem section viewed in the transverse plane. Inset shows a brightness-enhanced magnification of the labelled area in order to visualise cells retrogradely labelled with CTb. *E*, pooled distribution of retrograde CTb cell labelling in MAO (*n* = 3) following injections of CTb–saporin into the pyramis. Labelling plotted onto standard horizontal maps of the left and right MAO. Filled areas indicate regions in which cell labelling was observed in all 3 cases; hatched areas indicate total area occupied by labelled cells.

Post-mortem immunohistochemistry confirmed that the injection sites of CTb–saporin were located mainly in the lateral parts of the pyramis (arrows, Fig.6*C*). In the inferior olive, retrograde labelling (intact and lysed cells) was located predominantly within the caudal/middle medial accessory olive (MAO, arrow, Fig.6*D* and *E*). In Fig.6*E* the core olivary territory in which retrograde labelling was found in all three available cases is shaded in black. This region of middle MAO is known to provide climbing fibre projections to the cerebellar vermal A zone, including lateral parts of the pyramis (Apps, 1990). No labelling was found in any other parts of the olive except in one animal, in which sparse CTb labelling was also observed in the dorsal fold of the dorsal accessory olive (DAO). The reduction in freezing behaviour that was observed in these three animals was therefore not likely to be due to lesioning of neurones in the DAO or other parts of the olive, which provide climbing fibre input to more lateral regions of the cerebellar cortex (Apps, 1990; Sugihara & Shinoda, 2004). In addition, no CTb staining was observed in any part of the PAG, indicating that retrograde degeneration of the vlPAG was also not likely to be responsible for the reduction in fear-evoked freezing behaviour (data not shown).

Given the evidence provided above for a strong physiological connection linking vlPAG with the lateral pyramis and the likely involvement of the climbing fibre system, in a separate series of experiments we performed chemical subtotal lesion of the inferior olive, using the neurotoxin *trans*-crotononitrile (Seoane *et al*. 2005). TCN is known to cause degeneration of neurones mainly in the caudal/middle medial regions of MAO, although degeneration of neurones in other parts of the olive also occurs, including DAO and the principal olive. In normal rats, prior to administration of TCN or vehicle, neuronal activation of vlPAG with DLH significantly increased the mean peak-to-peak amplitude of the H-reflex by 61 ± 0.2% (*P* < 0.01, Fig.7*A*, open bars: prePAG *vs*. PAG). Subtotal lesion of the olive did not significantly alter baseline H-reflex amplitude (*n* = 4; *P* > 0.05; Fig.7*A*, pre-PAG: open *vs*. filled bars), indicating that under the present experimental conditions, the inferior olive does not have a tonic influence on α-motoneurone excitability. However, 3 h after administration of TCN when inferior olive climbing fibre transmission is abolished (Seoane *et al*. 2005) vlPAG activation failed to exert a facilitatory effect on H-reflex amplitude (*n* = 4; *P* > 0.05, Fig.7*A*, filled bars: pre-PAG *vs*. PAG). Sites of vlPAG neuronal activation are shown in Fig.7*B*. The results from Sham-treated animals (*n* = 5; Fig.7*A*, hatched bars) were similar to baseline data in that vlPAG neuronal activation increased the mean amplitude of the H-reflex (by 113 ± 0.52%; *P* < 0.01).

**Figure 7:**
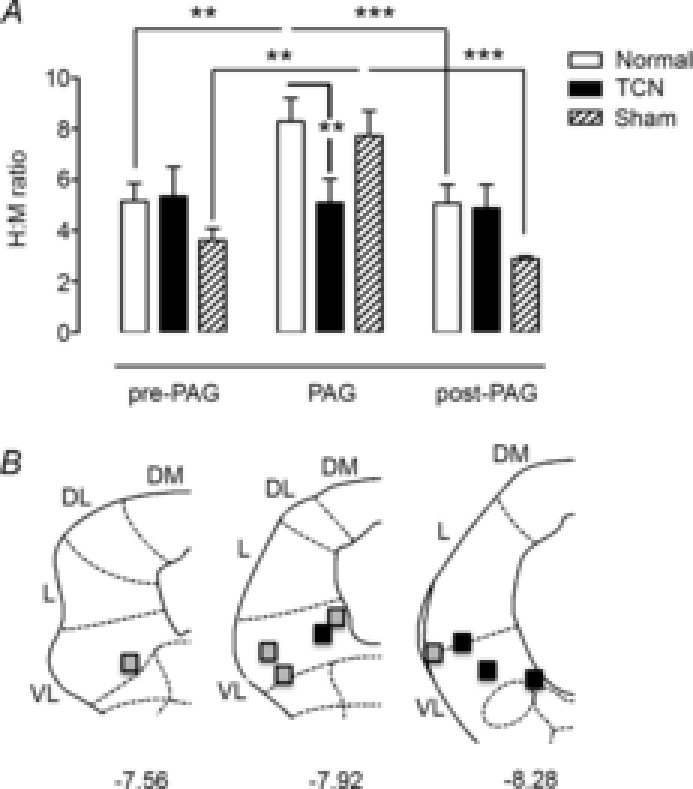
Inactivation of the caudal inferior olive abolishes the facilitatory influence of vlPAG activation on H-reflex amplitude *A*, H-response expressed relative to M-response (peak-to-peak amplitudes) before (pre-PAG), during (PAG) and after (post-PAG) vlPAG neuronal activation. Data for naive state (baseline), TCN-treated (*n* = 4) and Sham (*n* = 5) animals (**P < 0.01, Kruskal–Wallis with Dunn's multiple comparisons *post hoc* test). *B*, standard map of the left-hand PAG showing sites of chemical (DLH) stimulation for all available cases (*n *= 9). Coordinates are relative to bregma (DM, dorsomedial; DL, dorsolateral; L, lateral; VL, ventrolateral). Filled squares, TCN-treated animals; hatched squares, Sham animals.

Taken together, these lesion experiments therefore demonstrate that the vlPAG-mediated effect on spinal motor circuit excitability is dependent on the cerebellar pyramis, including associated climbing fibre pathways.

### Further consideration of the H-reflex data

Following vlPAG activation the *absolute* values of the H:M ratio between untreated (PAG H:M ratio = 6.0; Fig.5*B*) and CTb–saporin (PAG H:M ratio = 5.4; Fig.6*A*) treated animals are not significantly different (unpaired *t* test; *P *= 0.6). Additional examination of the data reveals that the statistical difference in the H:M ratio following vlPAG activation in untreated rats (control; Fig.5) is due to the lower average baseline value (prePAG H:M ratio = 4.4; Fig.5*B*) in comparison to a higher average baseline value for the CTb–saporin-treated group (prePAG H:M ratio = 5.1, Fig.6*A*). More specifically, following vlPAG activation the H:M ratio in the control group increased by ∼1.5-fold in contrast to a very modest increase of ∼0.3-fold in the CTb–saporin group. This *relative* difference is statistically significant (unpaired *t* test, *P *= 0.03; Fig.8*A*).

**Figure 8:**
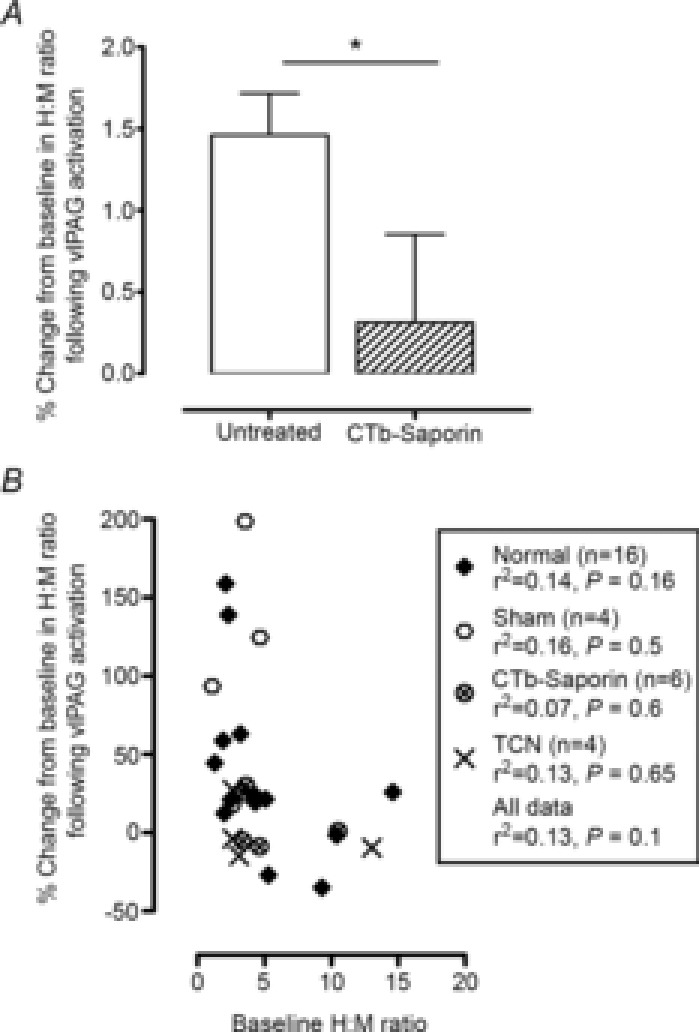
Further consideration of the H-reflex data *A*, group data showing that microinjections of CTb–saporin into superficial cortical layers of cerebellar vermal lobule VIII (pyramis) prevent the facilitatory influence of vlPAG activation on mean H:M ratio. Following vlPAG activation the H:M ratio in the untreated (normal, *n* = 16) group increased from its baseline by ∼1.5-fold in contrast to a very modest increase of ∼0.3-fold in the CTb–saporin-treated group (*n* = 6). This *relative* difference is statistically significant (unpaired *t* test, *P *= 0.03). *B*, following vlPAG activation there is no statistically significant correlation (*r*^2^ = 0.13, total *n* = 30; *P* > 0.05; Pearson correlation) between individual baseline H:M values and the corresponding percentage change in H:M ratio.

The intergroup discrepancy between baseline (prePAG) H:M ratio values arises presumably because of systematic differences in positioning of the percutaneous stimulating electrodes. This raises the possibility that variation between groups in the baseline values may be an important factor that accounts for our results. If this were the case then it might be expected that a correlation would be present between H:M ratio values before and after vlPAG activation which is independent of experimental group. Inspection of the average data in Fig.7*A* shows no such relationship.

In addition, on a case by case basis, there is no statistically significant correlation (*r*^2^ = 0.13, *n* = 30; *P* > 0.05; Fig.8*B*) between individual baseline values and the corresponding percentage change in H:M ratio following vlPAG activation. Thus, the differences between experimental groups in the effects of vlPAG activation on H-reflex excitability are not likely to be due to any differences between experimental groups in terms of baseline H:M ratio values.

## Discussion

Despite the fundamental importance of the vlPAG in coordinating and evoking freezing behaviour, little is known of the underlying neural pathways involved. Our findings provide novel insights into this issue, including: (i) demonstration of a strong physiological connection between vlPAG and the lateral cerebellar pyramis; (ii) the facilitatory influence of vlPAG on α-motoneurone excitability; and (iii) evidence that vlPAG-mediated effects on spinal motor function, and on fear-conditioned and innate freezing behaviour are dependent on the integrity of the input–output connectivity of this cerebellar region. It is important to emphasise that this does not exclude the possibility that the pyramis is also involved in other behaviours not tested in the present experiments. Nor does it exclude the possibility that other cerebellar cortical regions/pathways (and indeed other brain structures) may also be involved in freezing behaviour.

### Consideration of the results in relation to PAG function

Freezing immobility is a defence response elicited during passive–reactive coping, for example during a prey–predator encounter (Misslin, 2003). Although characterised by a cessation of voluntary movement, freezing is associated with increased muscle tone and a resultant fixed, tense posture. Such fear-induced immobility has been shown to be elicited by electrical stimulation of the ventrolateral and dorsolateral PAG (Vianna *et al*. 2001a) and reduced by vlPAG blockade (Monassi *et al*. 1999; Walker & Carrive, 2003).

By monitoring alterations in α-motoneurone excitability, the present results provide direct evidence that vlPAG activation can lead to the increased muscle tone that underlies freezing. This finding is, however, at odds with the ‘dual activation’ hypothesis of Walker & Carrive (2003), which proposes that the increase in muscle tone responsible for fear-induced freezing is *not* mediated by a direct effect of vlPAG on muscle tone, but instead by its interaction with other structures in the defence–arousal system. The hypothesis proposes that there are two components to the system. One component is thought to drive phasic movement (flight/fight), while the other generates tonic changes in posture (freezing). During conditioned fear, both components are thought to be activated along with vlPAG, which is postulated to have a strong ‘braking’ effect on the phasic component (preventing flight/fight), but only a weak ‘braking’ effect on the tonic component, so that freezing occurs, i.e. vlPAG activation is hypothesised to produce a modest *reduction* in muscle tone.

The present results found a strong positive correlation between freezing behaviour and vlPAG-induced increases in α-motoneuronal excitability (the final common output pathway from the CNS to skeletal muscles), which clearly does not support the dual activation hypothesis. Instead, our findings are consistent with the alternate view that freezing can be mediated by the vlPAG (Widdowson *et al*. 1986; Vianna *et al*. 2001a,*b*2001b; Leman *et al*. 2003).

### Consideration of the results in relation to climbing fibre function

The present study introduces the novel concept that the cerebellum plays an important intermediary role in vlPAG control of spinal motor function. This is supported by the increasing body of evidence to suggest that the cerebellum and in particular, its vermal compartment, is involved in emotional behaviours (Berntson & Torello, 1982; Schmahmann & Sherman, 1998; Strata *et al*. 2011). In particular, our results raise the possibility that vlPAG influence on freezing behaviour is dependent, at least partly, on the inferior olive climbing fibre system. Most theories of climbing fibre function consider them as ‘teaching’ signals important for cerebellar cortical plasticity (Yeo & Hesslow, 1998). However, it has long been known that olivary lesions severely disrupt muscle tone and cause postural abnormalities (Wilson & Magoun, 1945; King, 1948; Kennedy *et al*. 1982). Complex spikes generated by activity in climbing fibres can have both direct and indirect influences on cerebellar activity. For example, long lasting changes in Purkinje cell simple spike firing have been reported after complex spike activity (McDevitt *et al*. 1982), and ensemble encoding of complex spike activity has been shown to be related to motor output (Welsh *et al*. 1995; Welsh & Llinás, 1997; Blenkinsop & Lang, 2011). Although speculative, we propose that fear-induced activation of vlPAG drives ensemble activity in vermal olivo-cortico-nuclear circuits that, in turn, engage descending motor pathways (e.g. vestibulo- and reticulo-spinal paths) that are known to regulate muscle tone. There is substantial convergence of vestibulo- and reticulo-spinal pathways onto premotor interneurones in the mid lumbar cord (Davies & Edgley, 1994), which are associated with proximal, including anti-gravity musculature. As such, a lesion of climbing fibre projections that target the pyramis may induce enhanced simple spike firing (Cerminara & Rawson, 2004), which subsequently will inhibit activity in the descending pathways, thereby reducing muscle tone in postural muscles.

### Consideration of the results in relation to previous behavioural studies

Animals with lesions of the cerebellar pyramis displayed no detectable difference in tasks that assess levels of anxiety, notably performance in the open field and elevated plus maze. By contrast, the same lesions resulted in deficits in conditioned freezing behaviour. The deficit may be in motor learning and/or performance. Both autonomic (Supple & Leaton, 1990) and fear-related conditioning (Sacchetti *et al*. 2002, 2007) have previously been shown to require the integrity of the cerebellar vermis. In particular, Sacchetti and colleagues have shown in rats that parts of the cerebellar vermis are important sites of plasticity related to consolidation of conditioned fear memory (Sacchetti *et al*. 2009).

However, the results of Sacchetti *et al*. (2009) differ from our findings in at least three important respects. First, different cerebellar vermal lobules were implicated in fear conditioning in their study (lobules V/VI *vs*. lobule VIII in the present experiments); second, they found that mossy fibres but not climbing fibres were involved in consolidating the memory trace. And third, we found freezing deficits could also occur to an *unconditioned* fear stimulus, indicating that deficits in motor learning cannot fully explain our findings. While the present data do not exclude a role for mossy fibres, our results also emphasise the likely importance of climbing fibre projections to the pyramis. Specifically, the cortical location of evoked fields in the lateral vermis of lobule VIII, and the pattern of retrograde labelling in caudal medial accessory olive after CTb–saporin injections, identify the lateral A zone (Apps, 1990) and associated olivo-cortico-nuclear circuits as candidates for subserving conditioned and innate freezing behaviour. This is a region of the cerebellum known in cats to receive auditory and limb input (Huang & Liu, 1991).

Importantly, animals with lesions of the cerebellar pyramis displayed behaviour indistinguishable from normal in motor tests of general coordination, balance and muscle tone. This suggests that the reduction in fear-conditioned and innate freezing behaviour in the same animals cannot be readily explained by wide-ranging motor dysfunction and a general inability to elicit an increase in muscle tone. Similarly, no overt sensory deficits were apparent as judged by the lesioned animals’ ability to respond normally to an acoustic startle stimulus, their orientation to the auditory cue during fear conditioning, and directed sniffing towards the olfactory cue in the unconditioned fear protocol. This is consistent with the commonly accepted view that cerebellar damage does not alter primary sensory function (Bastian, 2011). Instead, the results point to a highly specific motor impairment, and suggest that interference with increased muscle tone linked with freezing behaviour occurs only under very specific stimulus conditions.

It is also noteworthy that lesioned animals increased their display of other defensive behaviours such as risk assessment. Given the partly independent subcortical/brainstem pathways identified for innate *versus* conditioned fear behaviour (Gross & Canteras, 2012), this raises the possibility that lesions of the cerebellar pyramis release pathways associated with risk assessment that otherwise are masked by normal pyramis function.

### Concluding comments

The present study provides insights into how a central component of the neural network associated with fear-related behaviour, namely the vlPAG, is dependent on cerebellar circuits of the pyramis in order to engage spinal motor systems to increase muscle tone associated with freezing behaviour. This cerebellar region is necessary for normal levels of expression of freezing behaviour elicited by innate and learned fearful stimuli, raising the possibility that the pyramis serves as a point of convergence for different survival networks in order to generate a highly specific motor output critical to survival. More generally, the results also emphasise the importance of identifying the appropriate behavioural context in order to examine reliably the contributions of specific cerebellar regions in motor control and other functions.

## Funding

This work was supported by a BBSRC research grant (BB/G012717/1) to B.M.L. and R.A., a Physiological Society Undergraduate training in *in vivo* sciences grant to S.K. and a Physiological Society Vacation Scholarship awarded to E.V.E. and S.K. J.J.C. was an MRC scholar.

## Additional information

### Author's present addresses

T. C. Watson: Neuroscience Paris Seine, Navigation memory & Aging Team, F-75005, Paris France (1) Sorbonne Universités, UPMC University of Paris 06, UMR-S 8246; (2) INSERM, UMR-S 1130; (3) CNRS, UMR 8246.

## Key points

- At the heart of the brain circuitry underlying fear behaviour is the periaqueductal grey (PAG).
- We address an important gap in understanding regarding the neural pathways and mechanisms that link the PAG to distinct patterns of motor response associated with survival behaviours.
- We identify a highly localised part of the cerebellum (lateral vermal lobule VIII, pyramis) as a key supraspinal node within a chain of connections that links the PAG to the spinal cord to elicit fear-evoked freezing behaviour.
- Expression of fear-evoked freezing behaviour, both conditioned and innate, is dependent on cerebellar pyramis neural input–output pathways.
- We also address an important controversy in the literature, namely whether or not ventrolateral PAG (vlPAG) increases muscle tone. We provide evidence that activation of the vlPAG causes an increase in α-motoneurone excitability, consistent with a role in generating muscle tone associated with fear-evoked freezing.

## Abbreviations

CS: conditioned stimulus
CTb: cholera toxin b
DAO: dorsal accessory olive
DLH: dl-homocysteic acid
MAO: medial accessory olive
PAG: periaqueductal grey
TCN: *trans*-crotononitrile
US: unconditioned stimulus
vlPAG: ventrolateral periaqueductal grey

## Supporting Information

The following supporting information is available in the online version of this article.

Video S1 Lesion of the cerbellar pyramis reduces freezing behaviour in response to associatively conditioned fear.

Video S2 Lesion of the cerebellar pyramis reduces innate freezing behaviour in response to cat odour.
